# Regional Variability in Survival for Patients Diagnosed with Selected Central Nervous System Tumours in Canada

**DOI:** 10.3390/curroncol31060234

**Published:** 2024-05-29

**Authors:** Yifan Wu, Emily V. Walker, Yan Yuan

**Affiliations:** 1 School of Public Health, University of Alberta, Edmonton, AB T6G 1C9, Canada; ywu8@ualberta.ca (Y.W.); emily.walker@albertahealthservices.ca (E.V.W.); 2 Precision Analytics, Cancer Research & Analytics, Cancer Care Alberta, Alberta Health Services, Edmonton, AB T5J 3C6, Canada

**Keywords:** CNS tumours, pediatric CNS tumours, hazard ratio, regional disparity

## Abstract

Canada’s decentralized healthcare system may lead to regional disparities in survival among Canadians diagnosed with central nervous system (CNS) tumours. We identified 50,670 patients diagnosed with a first-ever primary CNS tumour between 2008 and 2017 with follow-up until 31 December 2017. We selected the four highest incidence histologies and used proportional hazard regression to estimate hazard ratios (HRs) for five regions (British Columbia, Prairie Provinces, Ontario, Atlantic Provinces and the Territories), adjusting for sex, tumour behaviour and patient age. Ontario had the best survival profile for all histologies investigated. The Atlantic Provinces had the highest HR for glioblastoma (HR = 1.26, 95% CI: 1.18–1.35) and malignant glioma not otherwise specified (NOS) (Overall: HR = 1.87, 95% CI:1.43–2.43; Pediatric population: HR = 2.86, 95% CI: 1.28–6.39). For meningioma, the Territories had the highest HR (HR = 2.44, 95% CI: 1.09–5.45) followed by the Prairie Provinces (HR = 1.52, 95% CI: 1.38–1.67). For malignant unclassified tumours, the highest HRs were in British Columbia (HR = 1.45, 95% CI: 1.22–1.71) and the Atlantic Provinces (HR = 1.40, 95% CI: 1.13–1.74). There are regional differences in the survival of CNS patients at the population level for all four specific histological types of CNS tumours investigated. Factors contributing to these observed regional survival differences are unknown and warrant further investigation.

## 1. Introduction

Primary central nervous system (CNS) tumours constitute a diverse group of neoplasms. Malignant CNS tumours are estimated to account for 1.4% of all newly diagnosed cancers in Canada in 2022 [1]. However, their incidence is disproportionate to their morbidity and mortality [2,3]. Due to their anatomical location, CNS tumours, including non-malignant tumours, can interfere with vital brain functions and neurological processes [2,3,4,5]. This disruption can result in disability and a lower quality of life for patients. Additionally, there exists significant heterogeneity in patient prognoses depending on tumour histology, grade, location, size and molecular features as well as patient characteristics (e.g., age) and clinical symptoms [5,6,7,8,9,10]. These prognostic factors play important roles in determining the treatment and management of CNS tumours [11,12,13,14,15].

Between 2010 and 2015, about 35.7% of primary CNS tumours diagnosed in Canada were malignant [16]. Glioblastoma, meningioma and unclassified tumours were the most commonly diagnosed histologies during this period, with patients diagnosed with glioblastoma experiencing the worst survival across all age groups. While CNS tumours are the most prevalent solid tumour among children and adolescents [17,18], survival rates are considerably more favourable in younger populations compared to older age groups [16,19,20]. Recent literature also suggests that there has been a disproportionate increase in the incidence of males diagnosed with primary malignant CNS tumours relative to females, which is accompanied by a decreased survival rate at one year after diagnosis [21].

Since healthcare in Canada is primarily managed, organized and delivered at a provincial or territorial level, there may be potential differences in the availability and accessibility of healthcare services across the country [22,23]. Our previous Canadian study found regional differences in survival across malignant histology types for patients diagnosed between 1992 and 2008 in Canada [19]. In this study, we aim to investigate whether these regional differences in survival persist among Canadians diagnosed with selected high-incidence CNS tumour histologies between 2008 and 2017, while adjusting for sex, age and tumour behaviour. 

## 2. Methods

### 2.1. Data Sources and Tumour Classification

The Canadian Cancer Registry (CCR) is a population-based administrative database that compiles person-oriented primary tumour diagnosis data reported by provincial and territorial cancer registries to Statistics Canada [24]. We used CCR linked to death information data from the Canadian Vital Statistics Death Database and from income tax returns (T1 Personal Master File) to conduct our analysis. This dataset contains patient information on primary tumour diagnosis and vital status from 1 January 1992 to 31 December 2017 across Canada. Due to the lack of cancer diagnosis data for Quebec from 2010 onward in the CCR at the time of this study, none of the carried-out analyses included patients diagnosed with primary CNS tumours in Quebec. We accessed the data through the Research Data Centre (RDC) located at the University of Alberta, operating under the authorization of Statistics Canada. Using the topographical sites code of the International Classification of Disease for Oncology 3rd edition (ICD-O-3), primary CNS tumours are classified as tumours originating from the following: meninges (C70.0–C70.9); brain (C71.0–71.9); spinal cord, cranial nerves, and other parts of the CNS (C72.0–C72.9); pituitary and pineal glands (C75.1–C75.3); and olfactory tumours of the nasal cavity (C30.0 [restricted to histology codes 9522–9523]). Histology codes were categorized into major and specific histologies following the histopathological grouping scheme used by the Central Brain Tumour Registry of the United States (CBTRUS) [25,26]. We only included primary CNS tumours with the following ICD-O-3 behaviour codes: benign (0), uncertain whether benign or malignant/borderline malignancy (1), or malignant (3). Primary CNS tumours with a behaviour code of carcinoma in situ (2) were considered “not classifiable by CBTRUS” and were excluded from our analysis. For simplicity, we condensed the three original behaviour codes into two categories: malignant and non-malignant (including benign and uncertain).

### 2.2. Study Population

The study population consisted of patients diagnosed with primary CNS tumours across Canada between 2008 and 2017. We excluded patients with missing birth or death dates (*n* = 10) since the calculation of survival time relies on these vital pieces of information. Patients with death certificates or autopsy-only diagnoses were also omitted (*n* = 640) due to potential limitations in the accuracy or completeness of post-mortem diagnoses. Where patients had identical dates of diagnosis and death (*n* = 65), we adjusted the date of diagnosis to be one day before the date of death to prevent the exclusion of these patients from the survival analysis. For patients who presented with multiple primary CNS tumours, we used the first primary CNS tumour diagnosis. In addition, given that brain cancer is the most common solid tumour affecting children and adolescents [17,18], we also conducted a separate analysis to investigate regional differences in survival across Canada among a specific subpopulation: pediatric patients aged 0 to 15 years. For comparison, we also created a historical cohort of primary CNS tumours diagnosed across Canada between 1998 and 2007 with the same inclusion criteria.

#### 2.2.1. Pan-Age Cohort

For this cohort, we selected four histologies with the highest incidence rates across Canada: glioblastoma (GBM), malignant glioma not otherwise specified (NOS), meningioma and unclassified tumours [16,27]. Their corresponding ICD-O-3 histology and behaviour codes are provided in Supplementary Table S1. Although tumours of the cranial and spinal nerves also exhibited high incidence, patients with this histology experienced highly favourable survival rates. Therefore, we refrained from investigating this histology further. Patients diagnosed with malignant glioma NOS and unclassified tumours in the Territories were excluded from our analysis due to the small sample size, which prevented meaningful cross-regional comparisons.

#### 2.2.2. Pediatric Cohort

High-incidence CNS tumours among pediatric patients include malignant glioma NOS, embryonal tumours, pilocytic astrocytoma and unclassified tumours [16,27]. Nonetheless, our analysis was constrained to pediatric patients diagnosed with either malignant glioma NOS or embryonal tumours. This was because, among pediatric patients diagnosed with pilocytic astrocytoma or unclassified tumours, there was an insufficient number of patients at risk (fewer than 20), deaths (fewer than 5) or both across the different regions in Canada.

### 2.3. Variables

**Figure 1:**
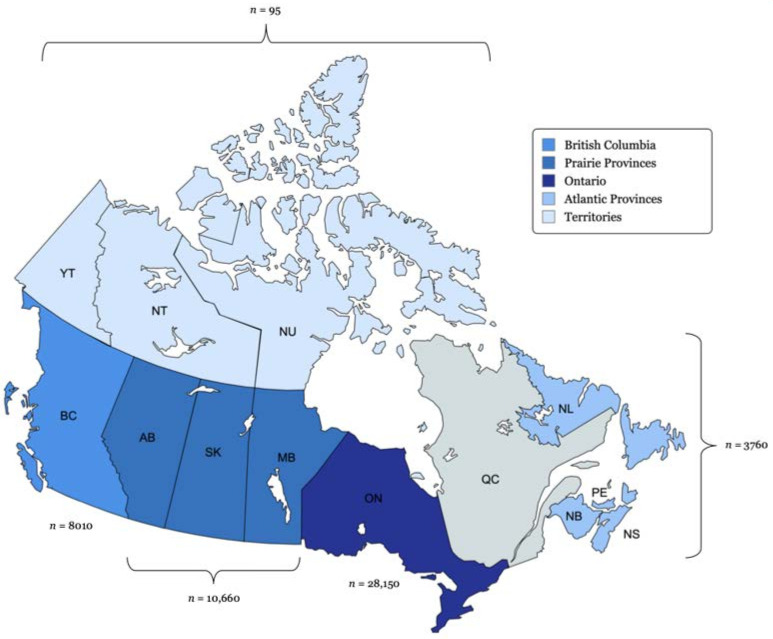
Geographical regions in Canada: Incidence of CNS tumours, 2008–2017. BC: British Columbia, AB: Alberta, SK: Saskatchewan, MB: Manitoba, ON: Ontario, QC: Quebec (data unavailable), NB: New Brunswick, NL: Newfoundland and Labrador, NS: Nova Scotia, PE: Prince Edward Island. *n*: total number of incident cases for each corresponding region from 2008 to 2017. Deeper shades represent higher population density.

The independent variable of interest is geographical regions in Canada, which includes the following: British Columbia, the Prairie Provinces (Alberta, Saskatchewan and Manitoba), Ontario, the Atlantic Provinces (New Brunswick, Newfoundland and Labrador, Nova Scotia and Prince Edward Island) and the Territories (Yukon, Northwest Territories and Nunavut). Provinces and territories were grouped together into broader regions due to the small sample size (Figure 1). Demographic independent variables include age at diagnosis (categorized into 0–4, 5–9, and subsequent 5-year intervals up to >85) and sex (female or male). However, due to an insufficient number of patients at risk (fewer than 20), deaths (fewer than 5), or both within some of the younger age groups for specific histologies, these age groups were aggregated for the purpose of analysis (meningioma: 0–29 years, unclassified tumours: 0–19 years). Among the selected histologies for analysis, only meningioma and unclassified tumours exhibit varying tumour behaviour, classified as either non-malignant or malignant. GBM and malignant glioma NOS are malignant by definition [18,28]. In our pediatric cohort, embryonal tumours were estimated to be 99% malignant [16].

### 2.4. Data Analysis

Our analysis necessitated the selection of high-incidence histologies and the aggregation of Canadian provinces and territories into Canadian regions. This strategic aggregation was driven by the dual imperative of meeting stringent vetting requirements set by the RDC when conducting survival analysis on a small number of events or patients at risk, and ensuring sufficient statistical power for our analysis. Five-year Kaplan–Meier (KM) estimates of overall survival were reported, stratified by sex, region, age group, tumour behaviour and selected histologies. We used Cox Proportional Hazards (PH) regression to estimate the hazard ratios (HRs) associated with region and sex for each of the selected histologies, adjusting for age group at diagnosis and tumour behaviour. The clearance cut-off date of 31 December 2017 was the censoring date. Since age at diagnosis did not meet the proportional hazard assumption for all selected histologies, it was used as a stratification variable in all Cox PH models. For meningioma and unclassified tumours, we also adjusted for tumour behaviour, and subsequently tested for potential effect modification between tumour behaviour and region, as well as between tumour behaviour and sex. Where there was evidence of important effect modification, HR estimates specific to the tumour behaviour strata were reported. We conducted all analyses using SAS version 9.4 (SAS Institute Inc., Cary, NC, USA) and STATA version 17 (StataCorp LLC, College Station, TX, USA).

### 2.5. Disclosure Rules and Rounding

To safeguard patient confidentiality, all reported frequencies and proportions underwent random rounding using an unbiased random rounding scheme with a base of five, in accordance with Statistics Canada vetting protocols. Human subjects research approval was granted by the Health Research Ethics Board of Alberta.

## 3. Results

### 3.1. All Histology Types

Between 2008 and 2017, 50,670 patients were diagnosed with a first-ever primary central nervous system (CNS) tumour in Canada, excluding Quebec. Table 1 summarizes demographic and tumour characteristic counts, proportions and five-year KM overall survival (OS) probabilities. A comparable number of males and females were diagnosed with primary CNS tumours, accounting for 47.5% and 52.5% of the cases, respectively. In total, 81.7% of CNS tumours were diagnosed in adults ages 40 years and above. Meningioma was the most frequently diagnosed histology. The largest proportion of patients were diagnosed in Ontario (55.6%), followed by the Prairie Provinces (21.0%), British Columbia (15.8%), the Atlantic Provinces (7.4%) and the Territories (0.2%). Patients diagnosed in Ontario had the highest unadjusted 5-year OS probability of 58.6% (95% CI: 57.9–59.2%). Conversely, patients in the Atlantic Provinces had the lowest unadjusted 5-year OS probability of 46.1% (95%. CI: 44.3–47.9%). The median follow-up time for all patients was 1.31 years. Specifically, for the analyzed histologies, the durations were as follows: 0.59 years for GBM, 1.03 years for malignant glioma NOS, 3.54 years for meningioma, 0.20 years for malignant unclassified tumours, and 2.92 years for pediatric patients with embryonal tumours.

### 3.2. Selected Histology Types

**Figure 2:**
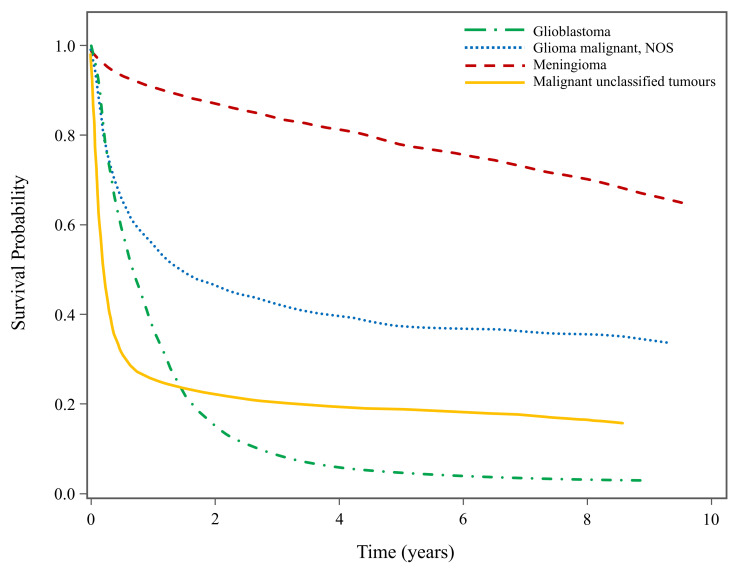
Histology-specific Kaplan–Meier survival curves for patients with selected high-incidence CNS tumours in Canada, 2008–2017 (excluding Quebec). NOS: not otherwise specified.

Among the selected histology types, patients diagnosed with GBM had the worst overall survival (5-year OS probability: 4.62%; 95% CI: 4.13–5.14), followed by malignant unclassified tumours (5-year OS probability: 18.83%; 95% CI: 16.81–20.93), malignant glioma NOS (5-year OS probability: 37.47%; 95% CI: 34.55–40.38) and meningioma (5-year OS probability: 77.94%; 95% CI: 77.05–78.80). Figure 2 presents the histology-specific KM survival curves for the selected histologies. Patients diagnosed with GBM had very poor prognoses, evidenced by the significant decrease in survival probability within the first year (1-year OS probability: 36.92%; 95% CI: 35.96–37.88) and subsequent second year post-diagnosis (2-year OS probability: 15.08%; 95% CI: 14.34–15.84). In contrast, patients diagnosed with meningioma exhibited a steady and modest decline in survival probability over time. Survival probability also decreased drastically within the first two years after diagnosis for patients with malignant glioma NOS and malignant unclassified tumours, although not to the same extent as GBM. Patients with malignant unclassified tumours experienced the fastest decline in survival probability within the first year of diagnosis. 

**Figure 3:**
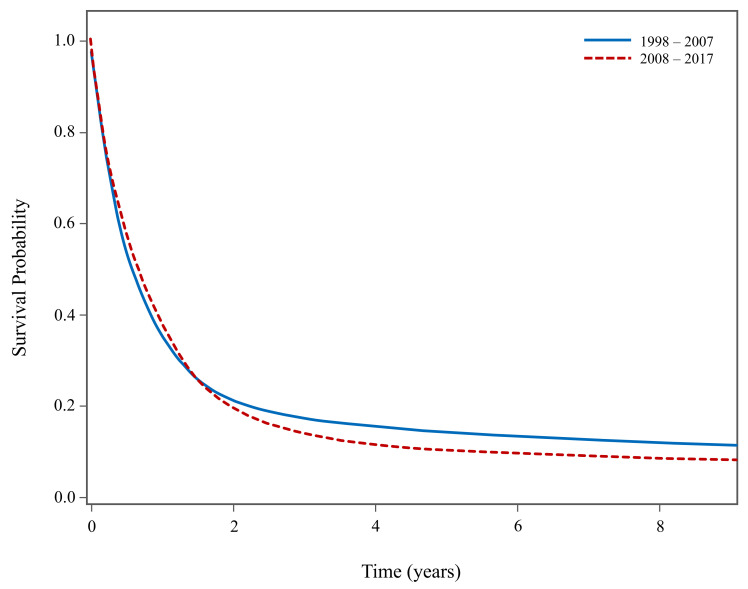
Kaplan–Meier survival curves for patients with selected malignant high-incidence CNS tumours in Canada between 1998–2007 and 2008–2017 (excluding Quebec). Does not include pediatric patients with embryonal tumours.

Figure 3 presents superimposed unadjusted KM survival curves for malignant tumours of the four histologies examined in our analysis (GBM, glioma NOS, meningioma and unclassified tumours), comparing patient survival from 1998–2007 to 2008–2017. Although Figure 3 suggests that patients diagnosed between 2008 and 2017 experienced poorer long-term survival compared to those diagnosed between 1998 and 2007, a closer examination revealed that this discrepancy is likely attributed to a higher proportion of patients diagnosed with GBM between 2008 and 2017 (78.2%) in contrast to 1998–2007 (64.4%).

#### Selected Histology Types for Pan-Age Cohort

**Figure 4:**
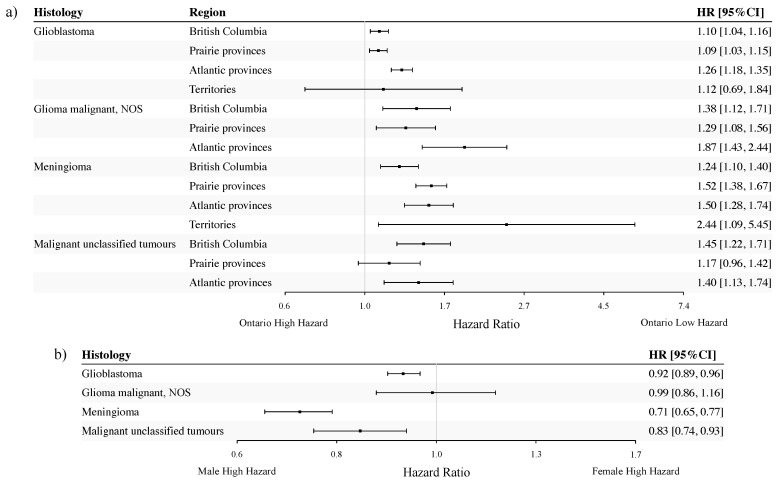
Histology-specific hazard ratios (HRs) for regions in Canada (**a**) and for sex (all regions combined) (**b**), adjusted for age at diagnosis and tumour behaviour (for meningioma only), 2008–2017. The reference groups were Ontario (**a**) and males (**b**). NOS: not otherwise specified.

Our analysis revealed effect modification between tumour behaviour and region (*p*-value < 0.0001), and tumour behaviour and sex (*p*-value = 0.097) for unclassified tumours. However, a closer inspection suggests that differential capture of non-malignant unclassified tumours between the regions may confound the regional HR estimates. Our previous studies showed that the Ontario Cancer Registry had the most comprehensive capture of non-malignant CNS tumours, with a disproportionally large proportion in the category of non-malignant unclassified tumours [27,29]. To avoid drawing erroneous conclusions due to the differential capture of non-malignant CNS tumours across regions in Canada, we restricted our subsequent analysis of unclassified tumours to malignant unclassified tumours. Thus, among the four selected histologies, three are malignant either by definition or by the imposed restriction. Tumour behaviour was included only in the Cox PH model for estimating meningioma-specific HR estimates (malignant vs. non-malignant meningioma adjusted HR = 3.08, 95% CI: 2.47–3.84). For each of the four histologies investigated in the pan-age cohort, adjusted HR estimates for region and sex are displayed in Figure 4a and Figure 4b, respectively. 

Overall, adjusted HRs for patients with CNS tumours showed similar variation across regions in Canada for the selected histologies investigated. In general, the highest HR point estimates were observed when comparing the Territories or the Atlantic Provinces to Ontario. The HR estimate for meningioma was the highest in the Territories, although it was accompanied by a wide confidence interval (HR = 2.44; 95% CI: 1.09–5.45). The Atlantic Provinces had the highest HR estimates for GBM (HR = 1.26; 95% CI: 1.18–1.35) and malignant glioma NOS (HR = 1.87; 95% CI: 1.43–2.44). For malignant unclassified tumours, HR estimates were similar between British Columbia (HR = 1.45; 95% CI: 1.22–1.71) and the Atlantic Provinces (HR = 1.40; 95% CI: 1.13–1.74).

For both GBM and malignant glioma NOS, the HR estimates were similar between British Columbia (HR = 1.10, 95% CI:1.04–1.16 for GBM and HR = 1.38, 95% CI: 1.12–1.71 for malignant glioma NOS) and the Prairie Provinces (HR = 1.09, 95% CI: 1.03–1.15 for GBM and HR = 1.29, 95% CI: 1.08–1.56 for malignant glioma NOS). However, the HR estimate for meningioma patients in the Prairie Provinces (HR = 1.52; 95% CI: 1.38–1.67) was notably higher than that for patients in British Columbia (HR = 1.24; 95% CI: 1.10–1.40).

Compared to males, females had a statistically significantly lower hazard of all-cause death for all histologies investigated except for malignant glioma NOS, for which their hazards were similar (Figure 4b). Notably, patients diagnosed with meningioma exhibited the most pronounced sex difference (HR: 0.71, 95% CI: 0.65–0.77), adjusting for tumour behaviour.

### 3.3. Selected Histology Types for Pediatric Cohort

**Figure 5:**
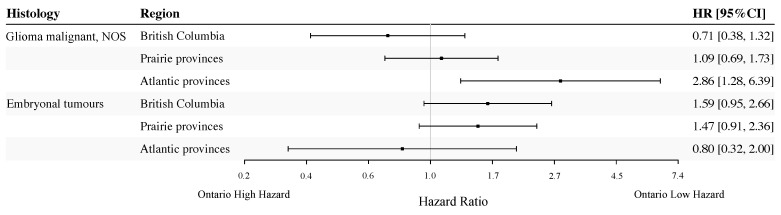
Histology-specific, sex- and age-adjusted hazard ratios (HRs) for regions in Canada for pediatric patients, 2008–2017. The reference group was Ontario. NOS: not otherwise specified.

Figure 5 presents age- and sex-adjusted HR estimates for different regions in Canada among pediatric patients diagnosed with either malignant glioma NOS or embryonal tumours. A statistically significant difference in survival was present when comparing Atlantic Provinces to Ontario for patients with malignant glioma NOS, although the observed difference was accompanied by a wide confidence interval due to the small sample size (HR = 2.86; 95% CI: 1.28–6.39). For embryonal tumours, there was a marginally significant higher hazard of all-cause death among pediatric patients in British Columbia (HR = 1.59; 95% CI: 0.95–2.66; *p* = 0.079) and the Prairie Provinces (HR = 1.47; 95% CI: 0.91–2.36; *p* = 0.11), when compared to Ontario. For both histologies, we did not find a statistically significant difference in survival between males and females in pediatric patients (*p*-value range: 0.46–0.55).

## 4. Discussion

Between 2008 and 2017, 50,670 people were diagnosed with a primary CNS tumour in Canada (excluding Quebec). The distribution of diagnoses across provinces/territories was consistent with the population size in each region, with the largest proportion in Ontario and the lowest in the Atlantic Provinces and Territories. Our analysis yielded evidence of variation in survival by region, with better survival outcomes in Ontario relative to all other regions. The largest effects were seen in the comparison between Ontario and the Territories for meningioma (HR = 2.44; 95% CI: 1.09–5.45), followed by the Atlantic Provinces for malignant glioma NOS (HR = 1.87; 95% CI: 1.43–2.44), British Columbia and the Atlantic Provinces for malignant unclassified tumours (BC: HR = 1.45; 95% CI: 1.22–1.7; Atlantic: HR = 1.40; 95% CI: 1.13–1.74), and the Atlantic Provinces for GBM (HR = 1.26; 95% CI: 1.18–1.35).

Regional variation in survival among patients with malignant primary CNS tumours was found previously in an analysis of CCR data from 1992 to 2008, with better survival among Ontario residents diagnosed with GBM, diffuse astrocytoma, and glioma NOS [19]. Several factors may explain the improved survival outcomes observed among patients in Ontario, the most populous region included in this analysis. Firstly, the higher population may lead to a more robust healthcare infrastructure, facilitating improved access to primary care for early diagnosis and specialized care for CNS tumours. Secondly, with a higher incidence expected in a larger population, there may be an increased volume of patients treated in the region, which may be associated with improved outcomes. Additionally, a larger patient population is more likely to attract research and clinical trials, which can yield valuable insights that can be seamlessly integrated into clinical practice. The impact of patient volumes, treatment at major academic centres, adherence to treatment guidelines and treatment by specialists on CNS tumour survival has predominantly been investigated for GBM. In a retrospective cohort study of 68,726 patients from the U.S. National Cancer Database from 2006 to 2013, the hazard of death among GBM patients treated at high-volume facilities was lower than that of patients treated at low-volume facilities (HR = 0.92; 95% CI: 0.89–0.94) [30]. Similarly, an analysis using the U.S. National Cancer Database from 2004 to 2013 found treatment at facilities in the highest quartile of patient volumes was associated with an 11–25% decrease in the hazard of death [31]. In a similar study, treatment at a high-volume academic facility was associated with the longest survival time [32]. Finally, in a case-control study of patients from 2006 to 2009 in the United Kingdom, survival time was compared across patients treated by general neurosurgeons or neurosurgeons with a specialty in neuro-oncology [33]. This study found evidence of increased survival among those treated by neuro-oncology specialists, along with a greater extent of tumour resection, reduced surgical deaths and shorter inpatient stays. Furthermore, it is important to highlight potential regional variations in preferred treatment approaches. For instance, between 2010 and 2019 in Ontario, approximately 68% of patients underwent surgery combined with chemoradiation or radiation therapy [34]. Conversely, chemoradiation, radiation therapy, or chemotherapy without surgery collectively accounted for only 9% of treatments received by patients. Similarly, in Alberta between 2008 and 2017, surgical resection was the preferred approach whenever deemed safe [35]. Biopsies were generally reserved for geriatric patients, those with multiple comorbidities, or those with tumours situated in deep, inaccessible locations. In contrast, treatment practices in the Atlantic provinces during this period were notably more conservative. Clinicians tended to opt for biopsy over surgical resection if they could not ensure a greater than 80% tumour resection [36]. However, further research is needed to better understand potential regional variations in treatment modalities for CNS tumours in Canada and their possible impact on patient survival, as current evidence is extremely limited.

Comparison with the United States may provide further evidence of a population-volume effect, where the 5-year OS probabilities are consistently lower in the Canadian cohort [37]. Nevertheless, making inferences solely based on population size, without the data necessary for a direct estimation of these effects, warrants cautious interpretation. More research is needed to determine whether these factors contribute to the survival differences seen across regions in Canada, and in particular between Ontario and the Atlantic Provinces and Territories. Additionally, the potential for the effects observed to be partially explained by residual confounding cannot be ruled out, especially in comparisons involving the Territories and Atlantic Provinces. There may be varying distributions of socio-demographic or lifestyle characteristics across regions that influence survival following a CNS tumour diagnosis. However, the data used for this analysis did not contain information on other potential confounders that were discussed above. Consequently, further research is needed to better understand the underlying reasons for the observed variation in survival across provinces/territories in Canada.

### Limitations

This analysis was subject to several limitations that should be considered when interpreting the results. First, there is potential variation in histology classifications across provinces/territories and over time, which may decrease the validity of inter-region comparisons [19]. This is particularly relevant considering the existence of related histopathological subtypes that represent varying grades and severities of tumours (for example, diffuse astrocytoma and GBM). For example, if Ontario has the tendency to classify tumours with histology codes that might be labelled as diffuse astrocytoma in other regions as GBM, this could account for the higher survival rate observed in Ontario. This aligns with findings from the United States, where the integration of tumour molecular profiles alongside traditional histopathology has led to shifts in tumour diagnoses, underscoring the dynamic nature of diagnostic classifications [38,39]. Specifically, a recent study utilizing data from the U.S. National Cancer Database from 2010 to 2015 examined tumour diagnosis discrepancies among patients with only histologically encoded classifications compared to those with integrated histologic and molecular diagnoses [38]. The findings revealed that 35% of histologically encoded anaplastic oligodendrogliomas underwent reclassification, with 10% of them being reclassified as GBM following the integration of molecular statuses into their diagnoses. Similarly, another study found that a sizeable proportion of anaplastic astrocytomas could be reclassified as GBM based on specific molecular profiles and hallmark alterations [39]. Reclassified GBMs exhibit slightly improved overall survival compared to GBMs that were not reclassified [40,41]. Although molecular biomarkers can aid in CNS tumour diagnoses, the accessibility of the technology needed to conduct such testing may not be uniform across Canada. Even when considering only histopathological diagnoses, studies in Europe and North America have consistently found high rates of interobserver variability in the diagnosis of glioma. This variability can result in misdiagnosis and inappropriate treatment regimens [42]. 

Furthermore, the categories of unclassified tumours and malignant glioma NOS are likely comprised of a heterogeneous mix of tumours with varying prognoses, limiting the inferences that can be drawn about the survival and relative survival of these groups across regions. Another limitation arose from sparse data in certain provinces/territories, which necessitated the amalgamation of certain provinces/territories into regions to attain sufficient statistical power for this analysis and, simultaneously, to comply with stringent RDC vetting rules. This merging may have inadvertently obscured any province- or territory-specific effects. Similarly, the limited number of histologies examined is recognized as a constraint, stemming from the rarity of CNS tumours and the need to adhere to RDC vetting rules. Residual confounding remains a concern in this analysis. Additionally, the lack of data on specific health system factors, including socio-demographic characteristics and treatment modalities, is an important limitation. Understanding the role of these unmeasured variables would provide more comprehensive insights into the factors underlying inter-regional differences in survival, as well as point to areas for improvement in different regions. Finally, the dataset’s clearance cut-off date of 31 December 2017, and the lack of available data from Quebec, constitutes a constraint in our analysis. Although this dataset represents the most recent release of the data, it is essential to recognize its temporal constraints. However, within these constraints, this population-based analysis of primary CNS tumours in Canada provides valuable insights into potential regional survival disparities, and highlights areas for further research to inform improvements in patient care and outcomes across the country.

## 5. Conclusions

This study provides a comprehensive examination of survival among primary CNS tumour patients in Canada between 2008 and 2017. We found evidence of regional variation in survival, with the most favourable survival outcomes in Ontario relative to all other regions, for all histological types analyzed. This was evident as all HR estimates were greater than one when comparing each region to Ontario (HR estimate range: 1.10–2.44), with the majority being statistically significant. These findings underscore the need for further research to better understand the underlying factors contributing to these disparities. In particular, the potential influence of healthcare infrastructure, treatment volumes and the role of specialists warrant further investigation. Such endeavours would provide a more comprehensive understanding of inter-regional survival differences and inform strategies for enhancing patient care and outcomes for primary CNS patients in Canada. 

## Figures and Tables

**Table 1:** Five-year Kaplan-Meier estimates of overall survival for patients diagnosed with central nervous system (CNS) tumours in Canada between 2008 and 2017 (excluding Quebec), stratified by demographics, tumour characteristics and selected histology types.

Variables	*n* (%) *N* = 50,670	5-Year Overall Survival (%) [95% CI]
**Sex**		
Male	24,055 (47.5%)	51.6 [50.9, 52.3]
Female	26,615 (52.5%)	62.3 [61.7, 63.0]
**Region**		
Ontario	28,150 (55.6%)	58.6 [57.9, 59.2]
British Columbia	8010 (15.8%)	57.0 [55.8, 58.2]
Prairie Provinces	10,660 (21.0%)	57.8 [56.8, 58.9]
Atlantic Provinces	3760 (7.4%)	46.1 [44.3, 47.9]
Territories	95 (0.2%)	57.2 [44.8, 67.9]
**Age group (years)**		
Pediatric (0–14)	2140 (4.2%)	79.2 [77.3, 81.0]
AYA (15–39)	7145 (14.1%)	85.8 [84.8, 86.7]
Adults (40–64)	21,315 (42.1%)	64.4 [63.7, 65.1]
Older adults (≥65)	20,070 (39.6%)	36.6 [35.9, 37.4]
**Tumour behaviour**		
Non-malignant	30,075 (59.4%)	78.8 [78.2, 79.3]
Malignant	20,595 (40.6%)	26.4 [25.7, 27.1]
**Selected histology ^a^**		
Glioblastoma	10,390 (20.5%)	4.6 [4.1, 5.1]
Glioma malignant, NOS	1250 (2.5%)	37.5 [34.6, 40.4]
Meningioma	12,000 (23.7%)	77.9 [77.1, 78.8]
Malignant unclassified tumours	1455 (2.9%)	18.8 [16.8, 20.9]
Embryonal tumour (pediatric)	355 (0.7%)	68.0 [62.7, 73.5] ^b^
**Total ^c^**	25,450 (50.2%)	42.7 [42.1, 43.4]

Note: columns and rows may not sum to totals due to rounding. AYA: adolescents and young adults. NOS: not otherwise specified. ^a^ Excludes patients diagnosed with the following histologies in the Territories due to small sample size: glioma malignant NOS, malignant unclassified tumours and pediatric patients with embryonal tumours. ^b^ Malignant embryonal tumours only. Note that ~99% of pediatric embryonal tumours were malignant [16]. ^c^ Total for selected histologies.

## Data Availability

Death-linked Canadian Cancer Registry data are available through the Research Data Centres in Canada upon data access application approval from Statistics Canada.

## Supplementary Materials

The following supporting information can be downloaded at: https://www.mdpi.com/article/10.3390/curroncol31060234/s1, Table S1: *International Classification of Diseases for Oncology*, 3rd Edition (ICD-O-3) histology and behaviour codes for selected histologies.
