# Supplementary material for: Regional Variability in Survival for Patients Diagnosed with Selected Central Nervous System Tumours in Canada
Source: Curr Oncol. 2024 May 29;31(6):3073–85. doi: 10.3390/curroncol31060234 (PMC11203179; doi:10.3390/curroncol31060234)
Supplement: Supplementary file 1 [file curroncol-31-00234-s001.zip › curroncol-2992768-supplementary.pdf]

Supplementary Table S1: International Classification of Diseases for Oncology, 3rd Edition (ICD-O-3) histology and behaviour codes for selected histologies.

Selected Histology	Histology / Behaviour Code
Glioblastoma	9440/3; 9441/3; 9442/3
Glioma malignant, NOS	9380/3
Meningioma	9530/0,1,3; 9531/0; 9532/0; 9533/0; 9534/0; 9537/0; 9538/1,3; 9539/1,3
Malignant unclassified tumours	9120/3; 9130/3; 9133/3; 9140/3; 8000/3; 8001/3; 8002/3; 8003/3; 8004/3; 8005/3; 8010/3; 8021/3; 8320/3; 8710/3; 8711/3; 8811/3; 8840/3; 8896/3; 8980/3; 9503/3; 9580/3
Embryonal tumour (pediatric)	8963/3; 9364/3; 9470-9474/3; 9480/3; 9490/3,0 9500-9502/3; 9508/3

Behaviour codes: 0 – benign, 1 – uncertain whether benign or malignant, 3 – malignant.
